# Leveraging existing provider networks in Europe to eliminate barriers to accessing opioid agonist maintenance therapies for Ukrainian refugees

**DOI:** 10.1371/journal.pgph.0002168

**Published:** 2023-07-13

**Authors:** Benjamin M. Nikitin, Daniel J. Bromberg, Lynn M. Madden, Heino Stöver, Robert Teltzrow, Frederick L. Altice

**Affiliations:** 1 Yale School of Medicine, New Haven, CT, United States of America; 2 APT Foundation, New Haven, CT, United States of America; 3 Frankfurt University of Applied Sciences, Frankfurt am Main, Germany; 4 Council of Europe, Strasbourg, France; Bielefeld University, GERMANY

## Abstract

Russia’s invasion of Ukraine caused a major refugee crisis, particularly impacting Central and Eastern Europe. Ukraine has one of the highest prevalence rates of opioid use disorder (OUD) in Europe, which increases the risk of HIV spread due to injection drug use. Opioid agonist maintenance therapies (OAMT) are a gold standard treatment for OUD and the prevention of HIV spread. Refugees who were displaced and previously maintained on OAMT in Ukraine require reliable care continuity, but OAMT is often highly regulated making it difficult to access. Using an implementation science lens, we sought to understand the barriers and facilitators that might impede OAMT continuity. We performed 23 semi-structured interviews with displaced patients with OUD and providers of OAMT and harm reduction. Interview participants were purposively sampled to include individuals from the highest-impacted countries: Poland, Germany, Czechia, Slovakia, Romania, and Hungary. Interviews focused on existing provider networks and barriers that refugees on OAMT faced during displacement. Though networks existed, there was little collaboration between providers and key stakeholders, such as NGOs, in overcoming barriers. Moreover, existing formal networks were not leveraged for rapid problem-solving. We found that despite existing networks, providers encountered substantial barriers to successfully coordinating access and retention in OAMT for refugees. Owing to insufficiently leveraged coordination between providers, clinics frequently turned patients away due to insufficient capacity, language barriers, and financial coverage issues. The limited geographic distribution of clinics in larger countries, such as Poland and Germany, further inhibited refugees from accessing and remaining on treatment. To support countries and providers in responding to a rapidly evolving crisis, collaborative learning combined with rapid cycle change projects used by the Network for the Improvement of Addiction Treatment (NIATx) model could be deployed to promote collaboration between providers both nationally and throughout the European Union to guide continuity of OAMT.

## Introduction

Russia’s invasion of Ukraine triggered a refugee crisis beginning in February 2022, with 10 million externally displaced persons leaving Ukraine for Europe and half returning to Ukraine as of January 2023 [1]. Ukraine has one of the highest numbers of people who inject drugs (PWID) in Europe, comprising roughly 1.4% of its total population [2]. Most (82%) of the 360,000 PWID in Ukraine have opioid use disorder (OUD) [3], as opioids are the most commonly injected drug in the country [4]. Non-medical use of opioids, especially with injection, has negative medical, psychiatric, and social consequences to individuals: overdose, death, endovascular and skin and soft tissue infections, and transmission of blood-borne viruses like HIV and HCV [5–7]. Ukraine has the second-highest HIV prevalence in Europe, after Russia, with 20.9% of PWID having HIV [8]. Wartime displacement of Ukrainian PWID across Europe can therefore lead to negative consequences if opioid agonist maintenance therapies (OAMT) with either methadone or buprenorphine are interrupted, potentially leading to overdose, suicide, or HIV outbreaks in Europe [9].

Maintenance with OAMT is the gold standard for treating OUD [5, 10]. OAMT reduces drug use, overdose, death [11], and transmission of HIV and HCV infections [12]. OAMT is often highly regulated and mostly prescribed daily, with varying levels of supervision depending on the stability of the patient [13]. Daily OAMT should not be interrupted and, if so, results in painful opioid withdrawal symptoms; relapse to opioid use; and high mortality from overdose, suicide, and HIV transmission–a painful lesson learned when Russia illegally annexed Crimea and abruptly stopped all OAMT [14]. Moreover, displacement-related trauma can increase the likelihood of risky drug behavior, making provision of OAMT among displaced Ukrainians highly important [15]. Prior to the Russian invasion of Ukraine, 17,232 individuals were on OAMT in governmental clinics in Ukraine [16], with thousands more in private clinics [17, 18]. Although all Western and Central European countries provide OAMT, each with differing regulations and delivery strategies, demand constraints from the refugee crisis may undermine the benefit to individual and public health. Ensuring efficient enrollment and retention in OAMT treatment of Ukrainians is therefore crucial to healthcare continuity, health and human rights, and limiting the spread of HIV across Europe [19, 20]. In this study, we aimed to explore the barriers and facilitators to accessing OAMT faced by displaced Ukrainian PWID and existing OAMT provider networks in European Union (EU) countries.

## Methods

### Context

Population estimates of the number of displaced Ukrainian OAMT patients currently in the EU are unavailable. However, as 90% of all refugees from Ukraine are women and children [21] while just 20% of OAMT patients in Ukraine are women [22], the number of displaced patients is likely to be relatively low. Still, because HIV is considered a disability that exempts adult men of conscription age (18 to 60) from staying in Ukraine, the number of displaced PWID and/or OAMT patients is likely still substantial [23, 24]. OAMT programs in many EU countries may be unprepared even for a minor influx of new patients seeking access. Any interruption of access and/or retention in OAMT treatment for Ukrainians with OUD represents a critical population-level health challenge for EU countries [19].

### Study design

This study was grounded in the *exploration* and *preparation* components of the Exploration-Preparation-Implementation-Sustainment (EPIS) framework. The EPIS framework was selected due to its focus on multi-level contextual factors across various stakeholders to lay the groundwork for the implementation and scale-up of OAMT, the best known evidence-based intervention for OUD [25]. As part of the *exploration* phase, we identified the most pressing health needs and priorities of Ukrainian refugees with OUD. Next, in the *preparation* phase, we applied the framework holistically to identify barriers and facilitators faced by key stakeholders and identify intersectoral solutions to these barriers. Guided by the EPIS framework, we designed the interview guides to assess both outer context factors (country-specific OAMT policies, networks between OAMT providers, collaboration between OAMT clinics and the government) and inner context factors (the perceived experience of patients and providers regarding the barriers they encountered). Based on the findings of this study, the remaining components of the EPIS framework, *implementation* of the identified intervention strategy and *sustainment* of that strategy, can be applied in subsequent studies [25].

### Sampling and study participants

To understand the inner context factors, we purposively sampled participants from six EU countries with the largest number of Ukrainian refugees: Poland, Germany, Czechia, Slovakia, Romania, and Hungary [1]. As a criterion for inclusion, all interviewed participants were either a) professional staff with experience with OAMT delivery, harm reduction services, or governmental health services within one of the studied EU countries or b) Ukrainian refugees who were on or seeking access to OAMT in an EU host country. Both staff and refugee participants were recruited by email and/or messaging apps and were encouraged to refer other stakeholders to the study. Participants did not have established relationships with the interviewer(s) prior to the study and were informed of the research goals prior to the start of each interview.

Semi-structured interviews (N = 23, 45 minutes each) were performed by two male coauthors (BMN and DJB, both fluent in English and Russian and trained extensively in qualitative research techniques) until data saturation was achieved. All interviews were performed in a private setting guided by the participant, either virtually or in-person. Interviews with professional staff were conducted in English, while interviews with patients were conducted in Russian, which all patients spoke fluently. No contacted individuals refused participation.

A semi-structured interview guide for professionals assessed two themes: 1) an understanding of and functionality of existing provider networks and 2) barriers and facilitators to OAMT access and continuation for displaced Ukrainians. Additionally, this guide asked about the quality of OAMT programs, services, and facilities, and did not collect personal or private information (S3 File). The interview guide for patients included questions regarding their chronological experience during their exodus from Ukraine (S4 File).

All interviews were transcribed verbatim. All Russian interview transcripts were subsequently translated to English and then back-translated to Russian to ensure cultural understanding of terminology, sayings, and metaphors to improve the accuracy of analysis [26]. OAMT coverage results for each country were compiled from European Monitoring Center for Drugs and Drug Action (EMCDDA) data [27], while perceptions about OAMT services were reported by participants.

### Data analysis

De-identified transcripts of translated interviews were coded using NVivo (Version 12.7.0) and analyzed using the EPIS framework through coding of inner and outer context factors [25]. Initial coding was deductive and used content from the interview guides, which led to iterative development of themes. These themes were subsequently mapped to the EPIS framework (i.e., inner vs. outer context). Coding was compared by BMN and DJB to evolve themes, and if disagreement emerged, an additional coauthor made the final decision. Interview quotations were extracted from transcripts to substantiate the analytic findings of the study.

### Ethics statement

This study was reviewed and approved by the institutional review boards (IRBs) of Yale University and the Ukrainian Institute on Public Health Policy. Both IRBs recommended verbal consent, as the study was deemed low risk; all participants were informed that their interviews would be de-identified and anonymous, stored securely, and used solely for research purposes that would not directly affect current/future healthcare provision. Participants were further asked not to provide any identifying information during the interview. Each interview was recorded and transcribed within 24 hours, and the original recording was immediately destroyed. In cases where a participant revealed identifying information, this information was not transcribed. De-identified transcripts and translations were stored on a secure Yale server. All provider interviews were conducted either virtually or in-person. All patients were interviewed using Zoom after they assured that they were in a private space. No participants were paid for their interviews or reimbursed for associated costs (e.g., internet use), as all prospective participants reported that they would not incur extraneous costs due to internet access when being interviewed.

## Results

In total, we interviewed 17 professionals from Poland (N = 7), Germany (N = 5), Czechia (N = 1), Slovakia (N = 2), Romania (N = 1), and Hungary (N = 1). Both direct care providers of harm reduction/OAMT (N = 11) and program administrators (N = 6) were interviewed. Of these participants, 10 were male and 7 were female. We also interviewed 6 patients, 2 each living in Poland, Germany, and Romania. Of these, 3 were male and 3 were female.

Several themes were developed during the analysis of these data. First, in their descriptions of existing collaborative networks, providers underlined that these networks were limited and minimally collaborative, with most being informally structured. Second, as identified by both patients and providers, there were substantial barriers to OAMT access for displaced Ukrainians.

### Existing networks

Countries have relied on both formal and informal networks to address emerging challenges related to OAMT and refugees. Networks of professionals in the EU involved in the delivery of OAMT exist to achieve a common goal: to provide treatment for OUD. These networks, however, are poorly characterized and require categorization as either *formal* or *informal*, which we derived inductively from data and categorized in this paper as such. We define formal networks as officially established professional groups that meet at least once a year and are aimed at information dissemination and/or resolution of logistical issues in the clinical setting. By contrast, informal networks are defined as unstructured interactions between providers and/or other officials that occur at irregular time intervals and/or are composed of undefined membership. Informal collaboration occurs on an as-needed basis (e.g., informing a provider that a patient is transferring from a different region). **Table 1** provides a detailed overview of these networks by country.

**Table 1:** Existing formal and informal networks and potential opportunities for OAMT service delivery improvement among European Union countries hosting the most Ukrainian refugees.

Country	Networks	Commonly Reported Barriers
**Poland** Number of refugees: 1.25M Estimated OAMT coverage: <20%	**Formal:** *Annual national conference* • A forum for discussing procedural and clinical issues relating to OAMT • Includes OAMT providers, harm reductionists, and government officials **Informal:** Contact between OAMT providers and the National Bureau for Drug Prevention • The National Bureau contacts providers on an as-needed basis to inform them of protocol/policy changes • E.g., at the start of the Russian invasion of Ukraine, a National Bureau official contacted every OAMT provider to notify them that Ukrainian refugees could be treated free of charge, and that providers would be reimbursed by the National Health Fund	**Language Barriers** • Translation of documents is reportedly required by some clinics before patients may receive treatment • Online OAMT database describing sites and their policies is only available in Polish **Geographic Constraints** • Most clinics are in larger cities, while many Ukrainians have been directed to settle in smaller towns • As of July 1, 2022 public transit is no longer free for displaced Ukrainians • Due to perceived stigma, many patients do not report that they need access to OAMT or antiretroviral therapy and are consequently directed by volunteers to smaller towns
**Germany** Number of refugees: 915,000 Estimated OAMT coverage: <60%	**Formal:** *German Society for Addiction Medicine (DGS)* • Umbrella organization of OAMT providers • Meets twice annually • Priorities include provider recruitment to address shortage and policy change (e.g., new payment structure for take-home OAMT) • Reportedly unsupportive of process-related changes in OAMT clinics *Akzept (Bundesverband für akzeptierende Drogenarbeit und humane Drogenpolitik)* • National harm reduction umbrella organization (hosting 60 organizations) • Hosts bi-annual conference on psycho-social aspects of OAMT (“National OAMT Conference”, funded by the Ministry of Health) *Association of Statutory Health Insurance Physicians* • Group of 16 organizations, one per state • Meets monthly • Runs “circle of quality workshops” (audits of 2% of all patients in the German healthcare system) to ensure that providers are following best practices; those who do not are financially penalized **Informal:** An ad hoc working group in Berlin • Composed of specialists in addiction medicine, infectious diseases, harm reductionists, etc. • Meets every other week • Funded and developed the creation of a website for international patient linkages to Berlin OAMT clinics, available in German, English, Ukrainian, Russian, French, and Spanish (https://add-in.berlin/)	**Capacity** • Provider shortages due to lack of financial incentives and previously restrictive OAMT protocols, leading providers to fear prosecution (such fears have been unfounded since 2017 when new, flexible guidelines for OAMT were introduced) **Language Barriers** • Many clinics have reportedly requested that patients procure an interpreter independently **Financial Coverage** • Backlog with residency registration for Ukrainians delays access to OAMT **Geographic Constraints** • Most clinics are in larger cities, but many Ukrainians have been directed to settle in smaller towns; due to perceived stigma, many patients neglect to report that they require access to OAMT or antiretroviral therapy and are consequently directed to smaller towns • Eastern and Southern Germany (esp. Bavaria) are reportedly more restrictive with OAMT access • Due to program demands, some patients have needed to travel for hundreds of kilometers multiple times per week to receive medications
**Czechia** Number of refugees: 401,000 Estimated OAMT coverage: <40%	**Formal:** *Addiction Treatment Forum* • A part of the national Government Council for Drug Policy Coordination Working Group • Meets monthly/bimonthly, with additional meetings during crisis • An open forum composed of government officials, NGO directors, and addiction specialists **Informal:** None reported	**Capacity** • Only 10% of all OAMT programs provide methadone; the remainder is buprenorphine/naloxone (while most Ukrainians on OAMT use methadone, necessitating a potentially unpleasant/differentially effective medication change) **Financial Coverage** • Buprenorphine/naloxone is not covered by all insurers
**Slovakia** Number of refugees: 87,000 Estimated OAMT coverage: N/A	**Formal:** None reported **Informal:** Inter-provider contact • Because only three clinics serve the entire country, contact between providers is relatively easy to facilitate • Providers contact one another on an as-needed basis	**Capacity** • Only three methadone clinics serve the entire country • Providers generally start patients on relatively low dosages • Although permitted, psychiatrists are reportedly reluctant to prescribe buprenorphine • Centers generally provide limited access to take-homes for most patient, although no restrictions prohibit them from doing so • Note: Despite perceived capacity issues, very few Ukrainians have sought OAMT in Slovakia
**Romania** Number of refugees: 84,000 Estimated OAMT coverage: <10%	**Formal:** None reported **Informal:** Contact between OAMT providers and the National Anti-Drug Agency • Providers can contact the Agency whenever there is an issue or the patient database needs to be accessed • E.g., When displaced Ukrainians in need of OAMT had challenges reaching Bucharest due to a car breakdown, a clinic contacted the National Anti-Drug Agency, who procured transport for them	**Capacity** • Only five OAMT clinics are available, all in Bucharest • Only methadone is available; buprenorphine is not • Pills only available up to 20 mg • Patients taking high doses need to take a very large quantity of pills • All centers are at maximum capacity and have frequently been unable to accommodate new patients **Language Barriers** • Most clinics do not have translators/interpreters who can help Ukrainian refugees **Financial Coverage** • Three of the five clinics are private; there is only partial insurance coverage for these services, if any • For those with insurance, methadone pills up to only 60 mg are covered **Geographic Constraints** • Long-distance travel is necessary for some patients, as OAMT is only available in the capital
**Hungary** Number of refugees: 27,000 Estimated OAMT coverage: <30%	**Formal:** *Hungarian Association on Addictions* • Hosts an annual conference for OAMT providers, addictologists, harm reductionists, etc. • The aim of the organization and its annual conference is the expansion and improvement of specialist training and skills • Process- and policy-related issues are discussed at the annual conference, but they have reportedly produced minimal alterations to the national protocol following each conference **Informal:** None reported	**Capacity** • Years-long waits are reportedly common • Low dosages of OAMT prescribed given due to restrictive policies **Language Barriers** • Communication is difficult as Hungarian is highly distinct from Ukrainian, Russian, English, and other languages spoken by refugees.

N/A (not available); NGO (non-governmental organization); OAMT (opioid agonist therapies); mg (milligrams); M (million)

#### Formal networks

We found that nearly all countries assessed have existing formal networks of OAMT providers locally and/or nationally. These groups, however, often meet infrequently and have low OAMT provider participation. For example, Poland has an annual conference attended by providers and members of the Polish National Bureau for Drug Prevention, but this conference is reportedly the only formal network for collaboration and problem-solving among providers.

*We organize something like a national conference*, *but we don’t have a platform for OST [opioid substitution treatment] … But we have a platform for harm reduction programs… where we meet annually*. (Ministry of Health official, Poland)

We found that many formal networks can benefit from additional structures and resources to leverage their meetings for process improvement. For example, several providers in Hungary met at an optional OAMT conference in May 2022 to discuss the need for a new national OAMT protocol to replace a long-expired one, but a process for developing the protocol had not been established since, according to participants.

*We don’t have a national… protocol… There was an OAMT conference… where doctors discussed that they need new guidelines*, *but I don’t know if any steps were made to renew these guidelines*. (Harm reduction provider, Hungary)

Formal networks are reportedly more organized in Czechia and Germany. In Germany, collaboratives exist on both a state and national level. In each German state, the Association of Statutory Health Insurance meets monthly to run “circle of quality workshops.” In these workshops, 2% of all confidential patient records are audited to ensure that OAMT providers and other physicians are acting in accordance with guidelines.

*We pick up about 2% of all the substitution patients and check them… We take them back and the doctor must report about them*. *And if it [the doctor’s report] is good—it’s good*. *But if it’s bad*, *he [the doctor] must pay or we take [away] permission [to treat] … If the patient takes cocaine*, *alcohol*, *and so on*, *being on substitution treatments and there is no response by the doctor*, *then that’s bad treatment*. *If I saw the documents*, *they are okay*: *I saw that he takes other drugs and we problematized it and he goes to the hospital to clear*, *then okay*, *that’s the right reaction*. (Harm reduction provider, Germany)

On the national level, the German Association for Addiction Medicine meets for a conference bi-annually. A substantial component of its work involves policy review and the recruitment of OAMT providers, which is essential for improved OAMT services.

#### Informal networks

Participants reported that providers occasionally meet informally with their colleagues and government officials to discuss process issues for OAMT delivery. An official at the Polish Ministry of Health described that, at the start of the war, she contacted every clinic in the country to inform it that OAMT would be covered by the state for all officially registered Ukrainian refugees.

*I assured them [public OAMT clinics] that they will get funding for this [treatment of Ukrainian refugees]*, *so they can give medicine to the patient*, *and they will be refunded afterward from our national insurance system*. (Ministry of Health official, Poland)

Two providers interviewed in Poland, however, implied that they were unaware of this communication from the Ministry of Health.

In Slovakia, because there are only three public methadone clinics in the entire country, providers meet frequently, albeit on an irregular basis, to discuss process-related issues.

*We don’t have a problem communicating between public methadone clinics*. *There are only three of them*, *so it’s not a problem*. (OAMT Provider, Slovakia)

During the refugee crisis, some informal collaboratives proved useful. For example, cooperation among European OAMT providers and public health experts (including volunteers from NGOs in Ukraine) enabled the development of a comprehensive database, *Treatment for Ukraine*, available in both English and Ukrainian, that guides refugees to OAMT and other harm reduction services.

### Observed barriers

Although some networks have attempted to eliminate barriers to OAMT access for Ukrainian refugees, many logistical and procedural issues remain, and there is no proactive process for solving them. Commonly identified barriers are outlined in **Table 1**.

#### Capacity

**Fig 1:**
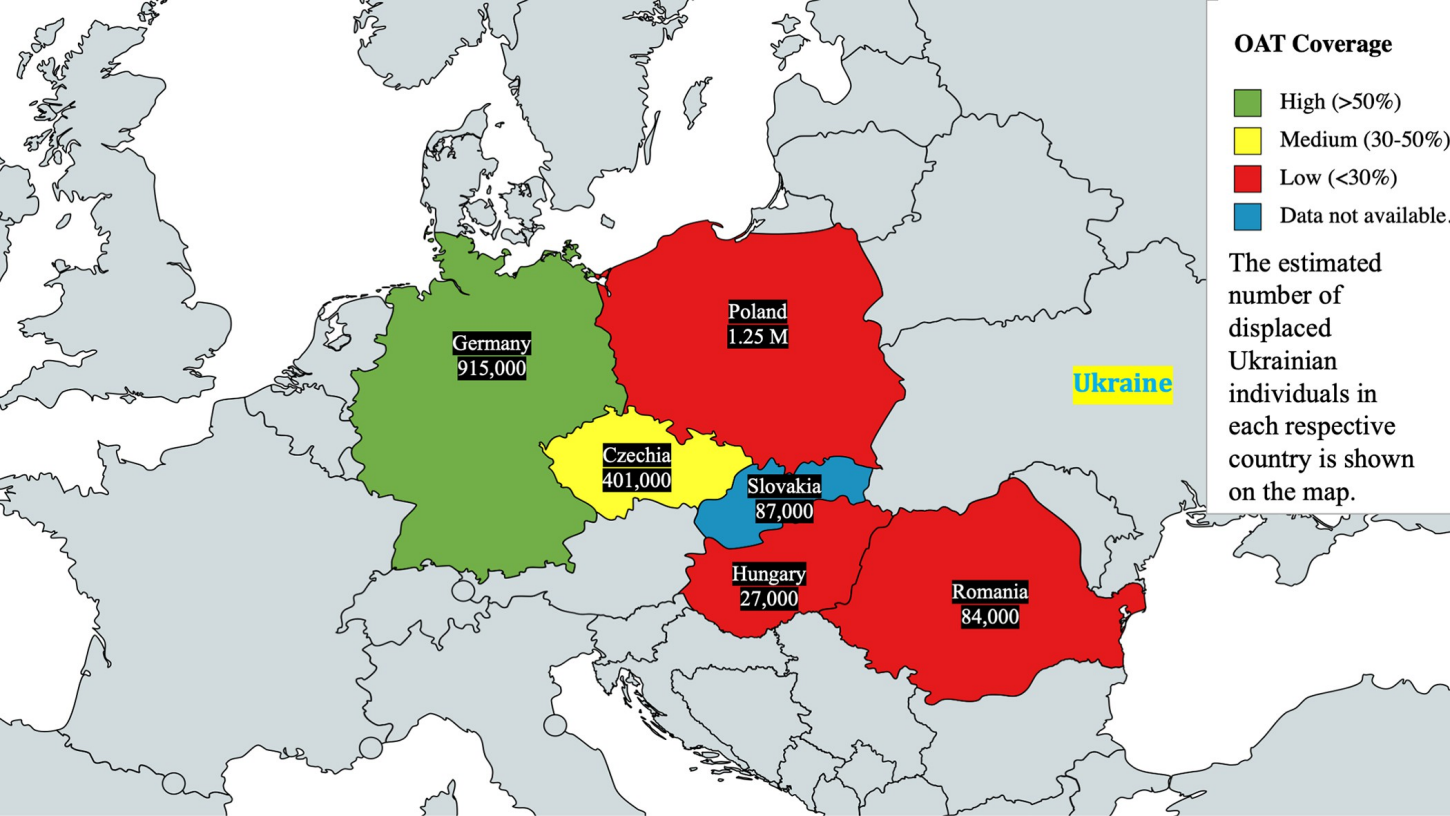
Map of OAMT coverage and number of displaced Ukrainians in studied European Union countries. Republished from MapChart with permission from Minas Giannekas, owner and founder of MapChart, original copyright 2023. Map base layer: https://www.mapchart.net/europe.html. Terms of use: https://creativecommons.org/licenses/by-sa/4.0/ (map labels were added after generation of map).

Even prior to the Ukrainian refugee crisis, many European countries had prolonged waiting lists to initiate OAMT. In general, OAMT coverage for high-risk opioid users ranged from less than 10% to over 50% (**Fig 1)**, with the lowest levels in countries bordering Ukraine (Romania and Hungary; data for Slovakia were unavailable) [1]. German interviewees noted a human resource shortage, with approximately 2,500 providers serving 127,500 PWID with OUD [27].

*We don’t have enough doctors who do OST [opioid substitution treatment]*. *And that’s the main issue… Nobody wants to take this “dirty” specialty*, *and nobody wants to take the patients*. (Harm reduction provider, Germany)

Facing heightened demand due to incoming refugees, OAMT clinics in Europe have struggled to expand intake capacity. In Romania, for example, demand for treatment was substantial even prior to the refugee crisis due to the high prevalence of PWID. Beyond high demand, access is critical, as the prevalence of HIV among PWID in Romania (16%) is one of the highest in Europe [27]. Combined with only 5 public OAMT clinics and low perceived capacity to treat a potential 20,000 high-risk opioid users, even a minimal influx from refugees requiring treatment poses perceived challenges for providers.

*The problem is that the resources we have are very low and I use the term ‘fortunate’ because we don’t have a bigger request [for OAMT from Ukrainian refugees]*. *Because if we did*, *I don’t know what we would do*. (OAMT provider, Romania)

#### Language barriers

OAMT programs in EU countries have not adapted their services to include languages spoken by Ukrainians. Although many countries have developed Ukrainian- and Russian-language hotlines for refugees seeking linkage to OAMT and/or harm reduction, patients reported that treatment centers lacked language services to assist with treatment enrollment. Because most staff at OAMT or harm reduction sites do not speak Ukrainian or Russian, some have reportedly requested displaced individuals to locate and hire their own interpreters. Due to stigma associated with OAMT and PWID, this has posed a substantial challenge for many patients.

*All the interpreters were volunteers… There were not enough interpreters [at the refugee center]*. *There was at most one*, *and then there was a lot of us [refugees]*, *and they were all juggling us*, *with not enough time*… *When she [a volunteer interpreter at the refugee center] heard that I was asking to translate something related to drugs*, *I mean methadone*, *she was like*, *“Is that drugs*, *illegal substances*?*” I said*, *“This is what the state gives out*, *the doctors give it to us*, *they recommend it so we can live a normal life” … And she says*, *“I’m with God*, *I go to church*. *I won’t translate that*, *I’m sorry*.*”* (Displaced Ukrainian OAMT patient, Germany)

Many clinics have also required Ukrainian refugees to pay for translation of their OAMT documents.

*I needed to translate the certificate into Polish because nothing would work with the Ukrainian certificate*. *And it turns out*, *that regarding the translation*, *I had further problems*. *Where to make a translation*? *… [I was told by a social worker] that I would have to pay for the translation*. (Displaced Ukrainian OAMT patient, Poland)

Refugees faced with the financial and logistical burden of securing translators and interpreters were delayed, and in some cases deterred, from accessing OAMT.

#### Financial constraints

Costs for OAMT in EU countries differ markedly by country, posing a major barrier for refugees who have limited resources. Displaced Ukrainians are required to register as temporary refugees prior to receiving any medical services, including OAMT; this may take several days, with reported bureaucratic backlogs delaying processing for several weeks to months. Delays of this length may put patients at risk of profound withdrawal symptoms.

*We needed health insurance [for OAMT]*, *and we got it in a day or two… That is at an accelerated pace*, *because everyone else had to wait a month*, *a month and a half*, *for all this registration [to be completed because] … There is a long queue*. (Displaced Ukrainian OAMT patient, Germany)

Furthermore, some OAMT options may be restricted and not covered by national insurers in EU countries. In Czechia, for example, some insurers do not support buprenorphine maintenance, yet Ukrainian OAMT patients may have been prescribed either methadone or buprenorphine. This results in patients either navigating transition to a new treatment or abandoning treatment altogether.

*In the Czech Republic*, *it’s nowadays a difficult situation because usually the medication you receive is not paid by health insurance*. *So that’s not true for methadone*, *methadone is free*, *but methadone is much more complicated to get… But if you have Suboxone (buprenorphine with naloxone in it) then it can generally be covered by health insurance*, *but it also depends on what insurance you have and other things*. *So sometimes it must be paid [out of pocket]*. (Harm reduction provider, Czech Republic)

#### Geographic constraints

Due to overpopulation, authorities often direct Ukrainian refugees to settle in less urban settings. As OAMT services are concentrated in larger cities (e.g., Berlin, Warsaw), refugees have encountered issues with access. Refugees stable on OAMT in Ukraine are often treated as new patients, initially necessitating daily supervised therapy with daily commutes to the nearest clinic, which can be up to several hundred kilometers.

*The first time*, *I got medication for one week*, *then I got it for two weeks*, *and then for two weeks again*. *And then*, *two weeks later*, *when I went there [to the OAMT clinic] again*, *I was supposed to be given medicine for three weeks*. *I told them that every time my trip takes at least a hundred kilometers… I mean*, *when you don’t have a car*, *it is a very long trip… Eventually*, *the third time*, *I just went with my wife and bought tickets for a high-speed train… And the third time I came there*, *I explained that it is very difficult to travel to you [the clinic]*, *I don’t know how it will be in the future*. (Displaced Ukrainian OAMT patient, Poland)

*[I must pick up OAMT medication] every day… It’s very far and very difficult*. *It’s about fifty minutes by bus to Augsburg*, *and from Augsburg also about an hour to Munich*. (Displaced Ukrainian OAMT patient, Germany)

In some settings, take-home dosing is either highly restricted or unprofitable for providers, placing extraordinary program-level demands on patients. Many patients have been dissuaded from continuing OAMT treatment due to long distances from clinics.

## Discussion

In the past decade, EU countries have experienced the largest mass migration of individuals since World War II [15]. Amid historic rates of displacement to EU countries in the past decade, challenges to access and retention in substance use treatment for displaced populations have been consistently reported, but solutions have been insufficiently deployed [15, 28]. To address the most recent refugee crisis from Ukraine, EU providers have individually attempted solutions, often in isolation and out-of-sync with strategies developed by neighboring colleagues. Although networks for collaboration between OAMT providers were identified in this study, our findings reflect that they were not optimally leveraged on the local or national levels for rapid problem-solving since the start of the refugee crisis. As the war persists, the identified barriers to OAMT access throughout the EU—capacity issues, language barriers, delayed financial coverage, and limited geographic coverage—should be collectively addressed to provide a haven for refugees who already experience extraordinary personal stressors and are predisposed to heightened drug use due to war-related trauma and adaptation to a new environment [15, 29].

These efforts will advance equitable healthcare to at-risk refugees, as outlined by the WHO Health 2020 plan [30] and Action 29 of the EU Drugs Action Plan 2021–2025 [20]; access to OAMT and retention in treatment will substantially improve the health of displaced PWID and minimize the risk of overdose and HIV transmission across Europe. Moreover, it is crucial for European OAMT systems to apply lessons learned from the mass movement of Ukrainian people across European borders to enhance preparedness for future refugee crises or other disruptions, such as climate-change-related natural disasters, that may lead to similar barriers [31].

Compared to the issues described in this study, highly similar challenges have been reported for general healthcare access among displaced populations in Europe. Rapid linkage of patients to both public and private healthcare systems has been a consistent challenge for refugee populations due to delayed financial coverage and strains on system capacity [32, 33]. Language barriers have also been pronounced; a 2012 study found that 40% of European health services lacked reliable access to language interpretation for patients and 54% lacked personnel with backgrounds in treating displaced populations, indicating that language-accessible, trauma-informed care for refugees is highly limited across Europe [34]. These barriers have re-emerged with the Ukrainian refugee crisis. For example, the influx of patients due to the crisis strained both primary and secondary health systems in Poland, with increased demand for treatment of non-communicable chronic conditions and infectious diseases as well as limited interpretation services [35]. To address these barriers, rapid deployment of solutions throughout the healthcare system is critical.

Though the barriers identified in this study are similar to those encountered throughout the European health system for migrants, a specialized focus is needed for OAMT, which has dire consequences if not continued for more than 24 hours. Moreover, in most EU countries, the OAMT system is not generally integrated with the rest of the healthcare system. Rather, it stands alone, with separate payment structures and entry requirements, which complicates financial coverage [36]. Furthermore, relative to barriers observed for displaced populations in the general healthcare system, challenges for displaced PWID are exacerbated due to stigma and OAMT clinics operating differently from other healthcare systems [37]. We hypothesize that the barriers described in this study are significantly more pronounced for OAMT access relative to other health sectors. For example, though geographic barriers complicate access to other secondary care in Europe [38], OAMT clinic distribution is even more sparse and requires more frequent patient visits. This makes geographic barriers substantially more complex. A focused effort by OAMT and harm reduction provider networks to improve access to their services is therefore necessary to address the challenges faced by displaced Ukrainian OAMT patients. This effort is particularly pressing, as neighboring EU countries lack a comprehensive infrastructure for delivering OAMT and HIV treatment due to substantially lower incidence of OUD and HIV in EU countries relative to Ukraine [9].

One potential solution that evolved during the interpretation of the data was that existing (and potentially new) networks could be leveraged in a time of rapidly evolving crisis. Although many of the EU countries in this study reported existing networks aimed at process improvement, most of these collaboratives were run pluralistically by OAMT and harm reduction providers without an evidence-based bundle of tools or facilitated coaching to guide improvements. Providers often did not prioritize system-level changes, thus limiting their ability to identify and resolve barriers. Studies have shown that focusing collaboratives on more solutions-oriented activities through guidance from a trained coach can substantially improve barrier elimination; effective facilitation contributes most effectively to process improvements [39, 40].

The Network for the Improvement of Addiction Treatment (NIATx) model provides a potential framework for achieving these goals. NIATx is composed of a bundle of implementation tools, like collaborative learning through coaching, that can be applied to existing networks. Collaborative learning, the practice of regularly meeting with and learning from other experts, can be a successful method for producing a more cohesive and effective response for removing barriers to treatment and supporting OAMT initiation/continuation [41]. In healthcare, the deployment of collaborative learning frameworks can have a major impact on institutions’ capacity to continue providing accessible and enduring care amid disruptor events, such as the present Ukrainian refugee crisis [42]. Crucially, multi-sectoral participation in collaborative learning can more efficiently expand and disseminate knowledge across stakeholders. In crises, healthcare networks are particularly interdependent on a wide range of stakeholders, including healthcare providers, service organizations, and the government. Therefore, solutions-oriented collaboration between these groups is critical [43]. Implementation of collaborative learning is consistent with recommendations by the Council of Europe for ensuring a more robust response to OUD treatment among displaced populations, but such efforts have been limited to date [15]. Though several other collaborative learning frameworks have been successfully deployed in the healthcare setting, including the Community of Practice and Extension for Community Healthcare Outcomes (ECHO) models, NIATx is the only model to our knowledge that is specifically designed for implementation in the addiction treatment setting [44].

When implemented through evidence-based models that target knowledge-sharing, NIATx could be a highly cost-effective method for resolving the identified barriers in this study [28]. For example, to expand capacity within OAMT clinics, providers could collectively coordinate reallocation of resources [45]. Collaboration could also enable better patient tracking to ensure consistent access to OAMT services [28]. Language barriers could be addressed through solutions-oriented meetings to learn lessons from other providers, such as the implementation of *ad hoc* professional or qualified volunteer interpretation services or linkage to comprehensive multilingual hotlines [28, 46, 47]. International collaboration between providers could alternatively enable the development of an EU-wide, digital certificate for treatment enrollment that translates to the receiver’s language, similar to the COVID-19 vaccine certificate [48]. To address financial barriers, providers could problem-solve methods of fast-tracking through vouchers to better accommodate displaced persons, particularly those at risk of withdrawal, as well as methods for back-dating eligibility for payment to avoid treatment gaps [49]. Finally, to remove geographic constraints in a crisis, collaborative learning could be applied to develop low-demand services like take-home medication for patients who were stable on OAMT in Ukraine [50].

As the EU allows considerable latitude to member states in domestic health matters, development of learning collaboratives might be most effective on the national level initially. The collaborative efforts between the German Society for Addiction Medicine (DGS) and the Ministry of Health to reform restrictive national OAMT policies provide a template for the formation and coordination of future networks. Through the collaboration spearheaded by the German Ministry of Health, DGS and government officials successfully identified necessary reforms to existing federal legislation. Consequently, in 2017, an amended Narcotics Prescription Ordinance was passed with reforms targeted at eliminating key barriers, including the physician shortage and lack of access to take-home medication [51]. Although collaboratives can be more rapidly developed on the national level, broader collaborations between EU countries, facilitated through groups like the EMCDDA Reitox focal point network [52], may achieve even more comprehensive improvements through international idea-sharing.

### Strengths/Limitations

This study contributes novel findings to the highly limited literature on HIV prevention for displaced populations. Through guidance from the EPIS framework to examine outer context policies and incorporate a purposive, diverse sample of interviewees reflective of the inner context (patients, providers of OAMT, harm reduction, and government services), our findings reflect a multistakeholder perspective of OAMT barriers and existing networks in action. We include key professional stakeholders of OAMT delivery in their respective countries to inform our findings, reflecting expert opinion.

Despite these novel findings, the study is not without limitations. Due to its qualitative nature, the study’s results reflect a situational analysis at the time of the interviews and hypothesis generation. They do not, however, provide an estimate or rank of the importance of barriers identified—which can be done through a mixed methods strategy like nominal group technique, one of the implementation tools used in NIATx [41, 53, 54].

Furthermore, as our reported findings are based on just 23 interviews in 6 countries, we do not aim to fully identify all barriers or existing provider networks. Rather, this study offers a high-level categorization and description of networks and barriers that should be expanded on in future studies. Specifically, future studies should examine the implementation of NIATx in networks of varying sizes and types to evaluate the practical applications of collaborative learning in these networks.

Finally, this study does not focus on the similar structural and non-structural impediments for continuity of care for other conditions like HIV, where discontinuity of care results in viral replication, development of resistance mutations, and potential for transmission to others [55]. In these conditions as well, NIATx or other collaborative learning frameworks could also serve as useful implementation strategies.

## Conclusions

Displaced Ukrainians have encountered major barriers of OAMT access or continuation in neighboring EU countries, including limited clinic capacity, language barriers, delayed financial coverage, and limited geographic coverage. Existing provider networks, both formal and informal, have been insufficiently leveraged for the rapid deployment of solutions that can expand access to OAMT for refugees. Consequently, we propose the incorporation of the NIATx model into existing regional, national, and international networks to achieve more efficient and targeted problem-solving. As future displacement of PWID due to violent conflicts or climate change is likely, more robust OAMT provider networks, guided by collaborative learning frameworks like NIATx, can ensure that barriers to OAMT access are more efficiently addressed in EU countries.

## Supporting information

S1 File Inclusivity in global research questionnaire, PLOS. (PDF)

S2 File COREQ checklist. (PDF)

S3 File Interview guide: OAMT and harm reduction providers. (PDF)

S4 File Interview guide: Displaced Ukrainian OAMT patients. (PDF)
