# Supplementary material for: Leveraging existing provider networks in Europe to eliminate barriers to accessing opioid agonist maintenance therapies for Ukrainian refugees
Source: PLOS Glob Public Health. 2023 Jul 13;3(7):e0002168. doi: 10.1371/journal.pgph.0002168 (PMC10343058; doi:10.1371/journal.pgph.0002168)
Supplement: S3 File — (PDF) [file pgph.0002168.s003.pdf]

Interview Guide: OAT and Harm Reduction Providers

Preamble

We know how busy you must be and thank you for taking the time to bring us up to date with all the work you are doing. First, how are you doing personally?

System

Can you describe the current structure of your program?

How many displaced patients from Ukraine have entered the program?

How have you had to change the structure of this program in response to the influx of Ukrainian patients?

Have you had any supply and/or distribution issues as a result of the influx of these patients?

Community/Collaboration

Are there specific OAT clinics and/or refugee, humanitarian, or government organizations that you have been collaborating with to help and/or retain patients?

What kinds of support do you need in order to do your job better? Would collaboration with other groups help with this?

Would you say there is a stigma associated with programs such as OAT, ART, etc. in [country]? If so, how would you say it has influenced your program and treatment of displaced individuals?

Organization

Has your program been effective with respect to newly enrolled individuals from Ukraine? What improvements could be made?

What kinds of governmental or non-governmental organizations have you worked with to help you address the needs of Ukrainian refugees?

How many more people have been added to your program? What regions are they from?

Has the implementation of legislation granting displaced persons from Ukraine access to healthcare been successful with regard to your personal clinic? Have there been any administrative issues, and if so, have they since been resolved?

Overall, what is going well? *(Prompt, if necessary: What could be going better?)*

Staff

Have you added staff and/or volunteers since the war to address the influx of patients?

What does your average day look like for the program – the staff in the program? *(Prompt: What is it really like to be you right now?) How are you communicating with patients to let them know about picking up needles/syringes?*

Client

Has your program had any issue with patient retention? Have you remained in contact with the patients who have since left the program?

How many patients have entered waiting lists? How long have they had to spend on your waiting list?

What have you learned in responding to this emergency? *(Prompt: Would you do things differently than how they have evolved?)*
