# Supplementary material for: Leveraging existing provider networks in Europe to eliminate barriers to accessing opioid agonist maintenance therapies for Ukrainian refugees
Source: PLOS Glob Public Health. 2023 Jul 13;3(7):e0002168. doi: 10.1371/journal.pgph.0002168 (PMC10343058; doi:10.1371/journal.pgph.0002168)
Supplement: S4 File — (PDF) [file pgph.0002168.s004.pdf]

Interview Guide: Displaced Ukrainian OAT Patients

<u>Preamble:</u>

We can't imagine how challenging things must be right now. We know how difficult it must be to talk about your experience and thank you for taking the time to tell us your story.

<u>How did you get here?</u>

Could you tell us how you got here?

Why did you choose to come here specifically?

Who did you come with? (E.g., family, friends, etc)

<u>What has been your experience along your journey?</u>

Were you able to transport extra supplies (e.g., needles/syringes, condoms, etc.) and medication (e.g., methadone, ART, etc.) to your new host country? Did you run into any issues with transporting these items?

Did you receive support from any organizations along your journey?

<u>What has been your experience since you arrived here?</u>

Were you able to find an OAT program? How difficult was it for you to find it?

Did you run into any issues when signing up for the program?

Did you receive any form of support from any humanitarian organizations or specific clinics that have helped with settling in or anything else?

<u>Community</u>

What does your usual day here look like?

Where are you living right now? How are the people you're living with? Do you feel comfortable around them?

Has anyone treated you badly as a result of your participation in the OAT program?

<u>Plans</u>

What are your plans for the next few months? Are you considering moving somewhere?
